# Association between olanzapine and immune function in lung cancer patients with anxiety and depression: a retrospective cohort study of medical records

**DOI:** 10.3389/fpsyt.2025.1655599

**Published:** 2025-12-08

**Authors:** Shang Chen, Guizhong Huang, Sen Lei, Xiaojun Lin

**Affiliations:** 1 State Key Laboratory of Oncology in South China, Guangdong Provincial Clinical Research Center for Cancer, Sun Yat-sen University Cancer Center, Guangzhou, China; 2 Department of Oncology, Qingdao Municipal Hospital, Qingdao, China

**Keywords:** anxiety, depression, lung cancer, olanzapine, tumor immunity

## Abstract

**Background:**

Anxiety and depression are prevalent in cancer patients and often impair immune function, compromising antitumor responses. Anti-anxiety medications, such as olanzapine, show promise in alleviating psychological distress, potentially enhancing immune function in patients with malignant tumors.

**Objective:**

This study aims to investigate immunological alterations in lung cancer patients with anxiety and depression and evaluate the immunomodulatory effects of olanzapine in this population.

**Methods:**

We retrospectively analyzed data from 179 oncology patients at a single center. Of these, 38 lung cancer patients served as the control group, while 33 lung cancer patients, admitted under standard hospital conditions, received olanzapine therapy (treatment group) during hospitalization. We monitored complete blood count, blood biochemistry, and lymphocyte subsets (CD3, CD4, CD8, NK cells, B lymphocytes). Anxiety and depression were assessed using the Self-Rating Anxiety Scale (SAS) and Self-Rating Depression Scale (SDS).

**Results:**

Lung cancer patients with concurrent anxiety and depression exhibited elevated CD3, CD4, CD8, NK cell counts, and CD4/CD8 ratio, alongside reduced Neutrophil-to-Lymphocyte Ratio (NLR) (P = 0.044, 0.001, 0.022, 0.039, 0.007, 0.003). Those with both conditions had lower serum albumin compared to patients with depression alone (P = 0.016) or either condition independently (P = 0.015). Patients with anxiety and depression showed reduced CD3, CD8, and NK cell counts compared to those with single conditions (P = 0.005, 0.037, 0.018). Baseline SAS/SDS scores showed no significant differences between groups (P = 0.385, 0.603). Olanzapine treatment significantly increased CD3, CD4, and NK cell counts (P = 0.001 each), reduced NLR and B lymphocytes (P<0.001, 0.036), and elevated HDL cholesterol (P = 0.014) compared to controls. Post-treatment, the treatment group’s SDS scores decreased from 42.64 ± 6.32 to 37.06 ± 8.34 (P<0.001), and SAS scores dropped from 50.48 ± 12.94 to 43.61 ± 13.47 (P<0.001).

**Conclusion:**

Anxiety and depression impair immune function in lung cancer patients, while olanzapine enhances CD3, CD4, and NK cell activity and reduces psychological distress, suggesting its potential as an adjunct in cancer immunotherapy.

## Background

Cancer patients endure significant psychological stress while confronting the prospect of mortality, which remains both imminent and uncertain. Individuals with malignant cancer face considerable stress factors (1, 2). Healthcare workers regularly observe anxiety and depression as common negative emotional responses among patients with malignant tumors. These psychological conditions affect patients’ mental well-being, living standards, and may influence cancer cell progression, as well as long-term survival and quality of life (3). Statistical evidence demonstrates that the incidence of anxiety and depression among cancer patients exceeds that of the general population by 1–3 times, with rates ranging from 20% to 50% (4).

Depression and anxiety can significantly compromise immune function in patients with malignant tumors (5). Research has consistently shown that patients experiencing anxiety and depression demonstrate irregularities in immune cells and cytokine-mediated immunity, highlighting the essential role of these components in anxiety and depression development (6). Evidence indicates that depression and anxiety may impair immune function in these patients through hormone secretion, which may inhibit the body’s ability to detect and eliminate tumor cells via bodily fluids (5, 7). However, studies examining immune function in cancer patients with anxiety and depression have produced variable results, partly attributable to limited sample sizes and outcome variability. While lymphocyte subsets are widely used as biomarkers across clinical studies, inflammatory markers such as the Lymphocyte-to-Neutrophil Ratio (LNR) remain underutilized in assessing inflammation levels and immune status (5). In clinical practice, the SAS and SDS serve as primary tools for evaluating anxiety and depression in patients with malignant tumors (8, 9). These assessment scales are widely implemented to measure anxiety and depression levels among cancer patients throughout China.

Patients with malignant cancer frequently experience anxiety and/or depression, arising from uncertainty about disease prognosis, apprehension regarding their physical condition, treatment efficacy and costs, and an inevitable decline in functional capacity (10). Notably, lung cancer maintains the highest global incidence and mortality rates. Approximately 50% of patients receive their diagnosis at an advanced stage, with surgical resection serving as the primary treatment for early-stage cases (11–13). In advanced lung cancer, chemotherapy, radiotherapy, and particularly targeted therapy, constitute the principal treatment modalities. Although these treatments have demonstrated significant outcomes, their limited applicability and drug resistance substantially restrict their clinical effectiveness. Therefore, there exists an urgent need to investigate novel adjuvant therapies (12). During the past five years, immunotherapy has emerged as a widely adopted approach in anti-tumor therapy, representing an additional systemic treatment option following surgery, chemotherapy, and targeted therapy. Research indicates that pharmacological intervention may enhance immune function in patients with depression (14).

Treatment for emotional distress in cancer patients encompasses both nonpharmacological and pharmacological interventions. While non-pharmacological therapies show promise, their quantification and variable efficacy across individuals pose challenges for standardization and widespread clinical implementation. Pharmacotherapy has thus emerged as a significant intervention strategy for tumor patients experiencing anxiety and depression. Studies indicate that sertraline demonstrates effectiveness in managing mental disorders (15). Olanzapine, a novel antipsychotic medication, shows potential as an adjunctive treatment for cancer-related depression. This study administered olanzapine to cancer patients to evaluate its effects on immune function, specifically focusing on lung cancer patients experiencing anxiety and depression.

## Methods

### Study design

This investigation utilized retrospective, anonymized clinical data collected after obtaining patient consent for treatment and informed consent documentation. The research adhered to all applicable legal and ethical guidelines, including the Declaration of Helsinki and the protocols established by the local Institutional Review Board (IRB)- Ethics Committee of Qingdao Municipal Hospital. Clinical trial number: not applicable.

Olanzapine serves a primary role in managing chemotherapy-induced nausea and vomiting (CINV) for lung cancer patients. The National Comprehensive Cancer Network (NCCN) Antiemesis Guidelines incorporated olanzapine into several recommended protocols in their 2018 edition, with its significance further enhanced in the 2020 edition. While all treatments adhere to standardized patient care guidelines, the implementation and emphasis of olanzapine therapy varies among different medical groups within our hospital, influenced by individual physician experience and guideline interpretation. This paper presents a retrospective analysis of clinical data to examine these treatment variations and their distinctive patterns.

### Study subjects

**Figure 1:**
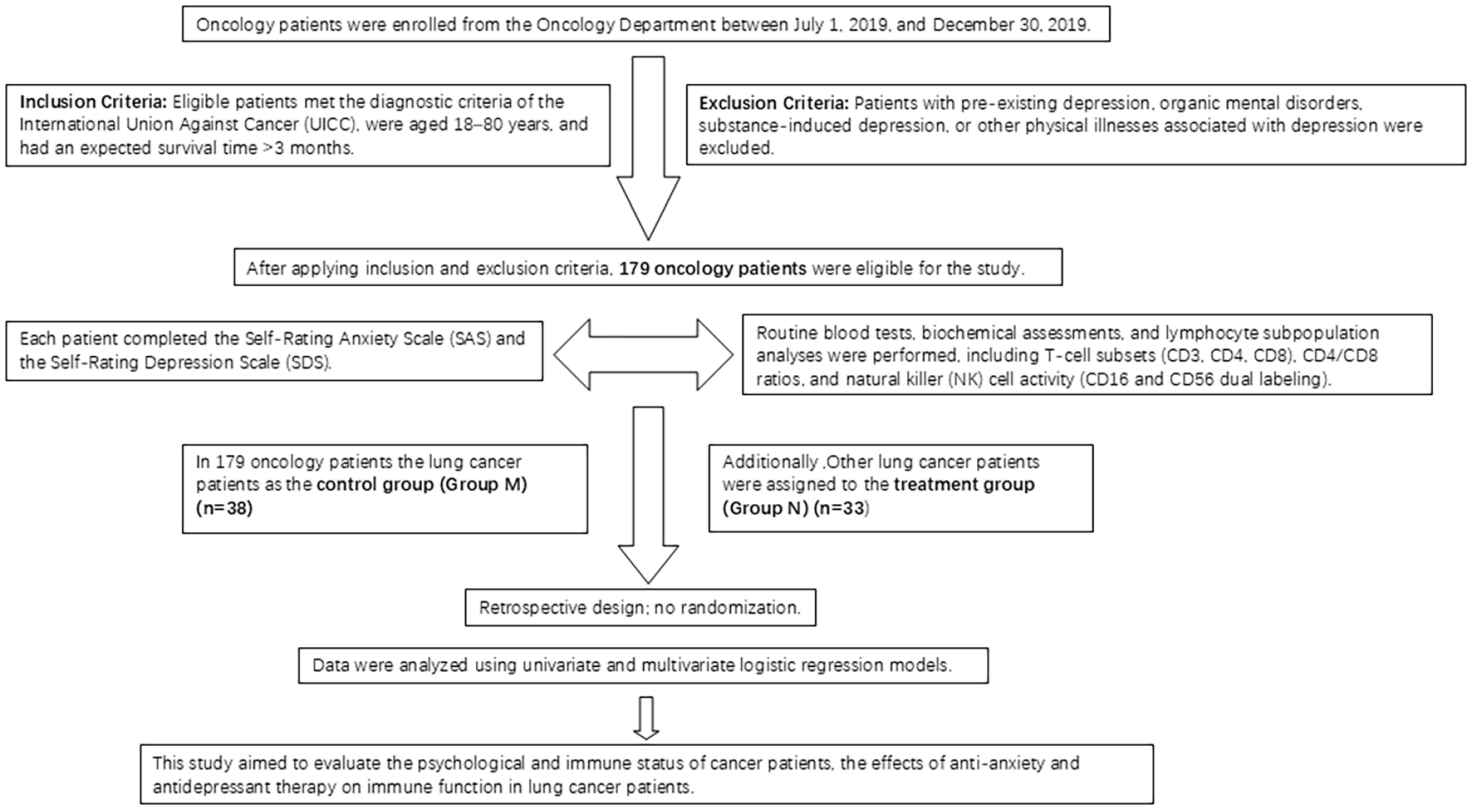
Flowchart of patient enrollment and study design.

The study included 179 patients admitted to the oncology department of Qingdao Municipal Hospital between July 1, 2019, and December 30, 2019. The patients were categorized into four groups: Group A (no anxiety or depression, 36 cases), Group B (pure depression, 108 cases), Group C (pure anxiety, 7 cases), and Group D (anxiety and depression, 28 cases). From the initial 179 patients, 38 lung cancer patients were selected as the control group (Group M). None of the patients in these groups were receiving olanzapine treatment. An additional 33 lung cancer patients were designated as the treatment group (Group N) (**Figure 1**).

### Inclusion criteria

Participants fulfilled the tumor diagnostic criteria established by the International Union Against Cancer (UICC) through pathological and/or cytological diagnosis. The study included individuals between 18 and 80 years of age with an expected survival duration exceeding 3 months.

### Exclusion criteria

The study excluded pregnant or lactating women and individuals with allergies. Additionally, participants with pre-existing depression prior to tumor diagnosis were ineligible. The exclusion criteria encompassed patients with organic mental disorders or depression induced by psychotropic substances and non-addictive agents. Individuals with other physical illnesses associated with depression were also excluded. The study did not include patients experiencing acute exacerbation of heart failure, respiratory failure, or other serious medical conditions. Participants exhibiting significant mental symptoms beyond depression and anxiety, or those with a history of mental disorders and positive family history, were deemed ineligible. The study excluded individuals who had undergone antidepressant medication and/or psychological therapy within 2 weeks before assessment. Participants lacking literacy skills or having only primary school education were not eligible.

### Data collection

The study included a cohort of 179 patients admitted to the oncology department between July 1, 2019, and December 30, 2019. Upon admission, standard examinations were conducted. Patients completed the SAS and the SDS to assess anxiety and depression symptoms. The examination protocol included routine blood tests, blood biochemistry, and lymphocyte subpopulations analysis (comprising T cell subsets using CD3, CD4, CD8, and CD4/CD8 ratios, along with NK cells using CD16 and CD56 dual labeling) utilizing the Epics-X L flow cytometer manufactured by Coulter in the United States. From the total cohort, 38 lung cancer patients were selected as the control group (designated as Group M).

Among the cohort, 33 lung cancer patients were allocated to the treatment group (designated as Group N) and received combined anti-anxiety and antidepressant therapy during hospitalization. The therapeutic regimen comprised oral administration of olanzapine (10 mg × 1 tablet) at a daily dosage of 10 mg over a 4-week period. Following the 4-week intervention, anxiety and depression symptoms were evaluated in patients with malignant tumors, along with measurements of routine blood parameters, blood biochemistry, and lymphocyte subpopulations specifically within the treatment group (Group N). Age, sex, and tumor stage were balanced between groups (**Tables 1**, **2**; all P > 0.05). Multivariate models did not include chemotherapy due to incomplete records.

**Table 1:** The distribution of anxiety and depression across different patient groups based on gender, age, tumor type, and cancer stage.

Group	A	B	C	D	X^2^	P
Gender						
male	18	57	5	16	1.25	0.74
female	18	51	2	12		
Age (years)						
<30	1	2	1	1	12.62	0.05
30-60	22	46	0	13		
>60	13	60	6	14		
Tumor type						
Lung Cancer	9	21	0	8	17.83	0.47
Digestive	9	37	5	11		
Gynecological	13	26	1	6		
Head and neck	3	6	0	2		
Lymphatic system	0	8	1	0		
Brain tumors	0	3	0	0		
Other	2	7	0	1		
stages						
I	2	6	0	3	10.69	0.30
II	8	15	3	3		
III	12	24	1	5		
IV	14	63	3	17		

(A group with neither anxiety nor depression; B with simple depression; C simple anxiety group; D anxiety and depression group)

**Table 2:** Comparison of general data of lung cancer patients in the M (control group) and N (treatment group, before drug treatment) groups.

Group	N(n=33)	M(n=38)	X^2^/F	P
Gender				
male	27	29	0.321	0.772
female	6	9		
Age (years)				
<65	14	24	3.052	0.099
≥.0	19	14		
stages				
I	0	1	1.272	0.736
II	2	3		
III	4	6		
IV	27	28		
Pathology				
Squamous carcinoma	6	7	2.930	0.403
adenocarcinoma	18	24		
SCLC	8	4		
Other	1	3		
SAS Score	42.63 ± 6.32	40.95 ± 9.39	0.875	0.385
SDS Score	50.48 ± 12.94	52.32 ± 16.13	-0.522	0.603

### Evaluation criteria

Routine Blood Tests, Blood Biochemistry, and Lymphocyte Subpopulations were analyzed according to standardized clinical criteria. For the SAS, the raw score was calculated by summing the scores of 20 items. The standard score was derived by multiplying the raw score by 1.25 and taking the integer portion. The SAS standard score classifications are as follows: 50–59 indicates mild anxiety, 60–69 indicates moderate anxiety, and 70 or above indicates severe anxiety. For the SDS, the raw score was similarly calculated by summing 20 items, with lower scores reflecting better mental state. The standard score calculation method matched that of SAS. An SDS standard score ≥50 indicates depressive symptoms, with the following classifications: 50–62 indicates mild depression, 63–72 indicates moderate depression, and scores above 72 indicate severe depression.

### Statistical methods

Statistical analyses were performed using SPSS 22.0 software. For normally distributed continuous data, the mean ± standard deviation was calculated. Between-group comparisons were conducted using the Student’s t-test. For non-normally distributed data, the Kruskal-Wallis H test was employed to compare medians across three or more independent groups, while the Mann-Whitney U test was utilized for comparisons between two independent groups. These data are presented as median (interquartile range) [M (P25, P75)]. Categorical data were expressed as proportions, with between-group comparisons conducted using the chi-square test (X² test). Statistical significance was defined as P < 0.05. Results are presented as mean ± standard deviation (SD). Group differences were evaluated using one-way analysis of variance (ANOVA) with subsequent *post hoc* comparisons. A p-value below 0.05 was considered statistically significant.

## Result

This study (**Table 1**) analyzed the distribution of anxiety and depression across patient groups categorized by gender, age, tumor type, and cancer stage. Patients were stratified into four distinct groups: Group A (neither anxiety nor depression), Group B (depression only), Group C (anxiety only), and Group D (both anxiety and depression). The gender distribution revealed that among males, Groups A, B, C, and D comprised 18, 57, 5, and 16 patients respectively, while females showed a distribution of 18, 51, 2, and 12 patients across the respective groups (X² = 1.25, P = 0.74). Age analysis indicated the following distribution: patients under 30 years numbered 1, 2, 1, and 1 in Groups A, B, C, and D respectively; those aged 30–60 years numbered 22, 46, 0, and 13; and those over 60 years numbered 13, 60, 6, and 14, respectively (X² = 12.62, P = 0.05). The tumor type distribution showed: lung cancer (9, 21, 0, 8), digestive system cancers (9, 37, 5, 11), gynecological cancers (13, 26, 1, 6), head and neck cancers (3, 6, 0, 2), lymphatic system cancers (0, 8, 1, 0), brain tumors (0, 3, 0, 0), and other types (2, 7, 0, 1) (X² = 17.83, P = 0.47). Cancer stage distribution revealed: stage I (2, 6, 0, 3), stage II (8, 15, 3, 3), stage III (12, 24, 1, 5), and stage IV (14, 63, 3, 17) (X² = 10.69, P = 0.30). This comprehensive analysis elucidates the relationships between anxiety and depression and various patient demographic and clinical parameters.

This study (**Table 3**) investigated the relationship between anxiety and depression and immune function in tumor patients by analyzing four distinct groups: Group A (no anxiety or depression), Group B (depression only), Group C (anxiety only), and Group D (both anxiety and depression). The NLR demonstrated significantly higher values in Group D (6.28 [3.16, 12.20]) compared to other groups (H (Kruskal-Wallis H test) = 13.64, P = 0.003). However, the platelet-to-lymphocyte ratio (PLR) showed no significant differences among groups (H = 3.417, P = 0.332), and similarly, the monocyte-to-lymphocyte ratio (MLR) remained comparable (H = 4.576, P = 0.206). Notable variations emerged in T-cell subsets: levels of CD3 (H = 2.783, P = 0.044), CD4 (H = 5.462, P = 0.001), and CD8 (H = 3.341, P = 0.022) were reduced in Group D, while the CD4/CD8 ratio exhibited significant variation across groups (H = 12.192, P = 0.007). Furthermore, NK cell counts were markedly lower in Group D (H = 2.885, P = 0.039). The analysis revealed no significant differences among groups in B cells (H = 1.434, P = 0.237), white blood cells (WBC) (H = 2.173, P = 0.093), Neu (H = 1.738, P = 0.161), Lym (H = 0.576, P = 0.699), hemoglobin (Hb) (H = 0.320, P = 0.811), albumin (ALB) (H = 2.567, P = 0.056), lactate dehydrogenase (LDH) (H = 0.486, P = 0.693), high-density lipoprotein cholesterol (HDL-C) (H = 0.455, P = 0.714), or Activated T lymphocytes(ATL) (H = 2.339, P = 0.077). These results suggest a significant association between anxiety and depression and altered immune function in tumor patients.

**Table 3:** Effects of anxiety and depression on immune function in tumor patients.

Group	A	B	C	D	H	P
NLR	3.15 (1.73, 7.09)	2.68 (1.67, 5.04)	5.91 (2.50, 8.26)	6.28 (3.16, 12.20)	13.64	0.003
PLR	168.00 (128.83,50.60)	161.76 (109.11,274.41)	115.83 (85.71,302.86)	139.75 (98.31,219.11)	3.417	0.332
MLR	0.28 (0.17,0.42)	0.31 (0.21,0.64)	0.36 (0.26,0.73)	0.22 (0.19,0.41)	4.576	0.206
CD3	69.84 ± 11.53	66.53 ± 14.18	64.46 ± 11.26	60.06 ± 12.90	2.783	0.044
CD4	45.28 ± 8.66	38.37 ± 11.91	38.38 ± 17.48	32.73 ± 13.29	5.462	0.001
CD8	26.79 ± 10.68	25.83 ± 9.51	20.46 ± 11.90	19.45 ± 10.04	3.341	0.022
CD4/CD8	1.60 (1.16,2.69)	1.44 (1.01, 2.58)	4.18 (1.12,4.18)	2.00 (1.27,2.86)	12.192	0.007
NK	23.31 ± 9.99	19.23 ± 9.43	18.45 ± 4.97	15.58 ± 12.20	2.885	0.039
B cell	9.82 ± 5.84	10.03 ± 7.10	5.13 ± 0.55	12.25 ± 7.02	1.434	0.237
WBC	6.55 ± 3.88	6.21 ± 3.71	7.99 ± 3.75	8.23 ± 5.03	2.173	0.093
Neu	4.79 ± 3.78	4.32 ± 3.53	5.74 ± 3.19	6.06 ± 4.89	1.738	0.161
Lym	1.27 ± 0.72	1.35 ± 1.54	1.51 ± 0.73	1.63 ± 0.76	0.576	0.699
Hb (g/L)	117.11 ± 20.32	117.25 ± 20.36	117.14 ± 21.53	113 ± 20.77	0.320	0.811
ALB	40.51 ± 3.99	37.10 ± 7.83	38.71 ± 8.65	36.00 ± 7.64	2.567	0.056
LDH	218.21 ± 43.03	222.83 ± 68.26	228.43 ± 79.13	36.67 ± 5.36	0.486	0.693
HDL-C	1.17 ± 0.28	1.10 ± 0.30	1.12 ± 0.44	1.12 ± 0.24	0.455	0.714
ATL	11.76 ± 6.53	13.65 ± 3.35	4.94 ± 1.49	12.07 ± 3.44	2.339	0.077

NLR (Neutrophil-to-Lymphocyte Ratio), PLR (Platelet-to-Lymphocyte Ratio), MLR (Monocyte-to-Lymphocyte Ratio), CD3(Cluster of Differentiation 3, %), CD4 (Cluster of Differentiation 4, %), CD8 (Cluster of Differentiation 8, %), NK (Natural Killer Cell, %), B cell (B lymphocytes, %),WBC(White Blood Cell, ×10⁹/L), Neu(Neutrophil Count, ×10⁹/L),Lym(Lymphocyte, ×10⁹/L), Hb(Hemoglobin, g/L),ALB (Albumin, g/L), LDH (Lactate Dehydrogenase, U/L),HDL-C(High-Density Lipoprotein Cholesterol, mmol/L), ATL(Activated T lymphocytes, %)

**Table 2** presents a comparative analysis of general data between lung cancer patients in the N (treatment group, before drug treatment) group (n=33) and the M (control group) group (n=38). The gender composition comprised 27 males and 6 females in the N group and 29 males and 9 females in the M group, showing no significant difference (X²=0.321, P = 0.772). In terms of age distribution, the N group included 14 patients under 65 and 19 patients aged 65 or older, while the M group had 24 patients under 65 and 14 patients aged 65 or older, with no statistical significance (X²=3.052, P = 0.099). The staging distribution demonstrated no significant differences, with stage IV being predominant in both groups (N: 27, M: 28, X²=1.272, P = 0.736). The pathological types, encompassing squamous carcinoma, adenocarcinoma, SCLC, and others, exhibited no significant differences between groups (X²=2.930, P = 0.403). The mean SAS score was 42.63 ± 6.32 in the N group and 40.95 ± 9.39 in the M group (F = 0.875, P = 0.385). The mean SDS score was 50.48 ± 12.94 in the N group and 52.32 ± 16.13 in the M group (F=-0.522, P = 0.603). These findings indicate no significant differences in general clinical data between the two groups prior to anti-anxiety and depression treatment.

**Table 4** presents a comparative analysis of immune function parameters between the N group (treatment group receiving anti-anxiety and depression therapy) and the M group (control group) in tumor patients. The NLR showed significantly lower levels in the treatment group (3.17 [1.82, 5.47]) compared to the control group (6.54 [3.55, 12.78]; Z=-3.851, P = 0.00). No significant differences were observed in PLR and MLR values between the groups. The treatment group demonstrated significantly elevated CD3 levels (69.41 ± 8.12) compared to the control group (55.17 ± 23.03; t=3.373, P = 0.001). Similarly, CD4 levels were significantly higher in the treatment group (43.27 ± 9.56) versus the control group (35.41 ± 8.26; t=-3.353, P = 0.001). CD8 levels and CD4/CD8 ratios remained comparable between groups. NK cell levels were significantly elevated in the treatment group (19.34 ± 5.66) compared to the control group (13.24 ± 8.20; t=3.352, P = 0.001). Conversely, B cell levels were significantly lower in the treatment group (8.88 ± 3.68) compared to the control group (11.72 ± 6.33; t=-2.147, P = 0.036). The analysis revealed no significant differences in WBC, Nc, Lc, HB, ALB, LDH, or ATL between groups. HDL-C levels were significantly higher in the treatment group (1.23 ± 0.30) compared to the control group (1.07 ± 0.25; t=2.535, P = 0.014). These results indicate that anti-anxiety and depression therapy was associated with alterations in specific immune function parameters.

**Table 4:** Effects of anti-anxiety and depression therapy on immune function of tumor patients Groups: M (control group) and N (treatment group).

Groups	N (treatment group)	M (control group)	Z/t	P
NLR	3.17(1.82,5.47)	6.54(3.55,12.78)	-3.851	0.00
PLR	153.55(117.91,240.20)	173.47(115.47,276.38)	-0.357	0.361
MLR	1.04(0.15,155.18)	0.61(0.24,118.41)	-0.054	0.479
CD3	69.41 ± 8.12	55.17 ± 23.03	3.373	0.001
CD4	43.27 ± 9.56	35.41 ± 8.26	-3.353	0.001
CD8	23.95 ± 5.16	21.41 ± 10.60	1.201	0.235
CD4/CD8	2.0(1.7,2.11)	2.04(1.52,3.00)	-0.664	0.253
NK	19.34 ± 5.66	13.24 ± 8.20	3.352	0.001
B cell	8.88 ± 3.68	11.72 ± 6.33	-2.147	0.036
WBC	8.29 ± 3.96	6.73 ± 3.34	1.798	0.077
Neu	6.12 ± 3.72	4.76 ± 3.25	1.644	0.105
Lym	1.72 ± 0.60	1.41 ± 0.81	1.803	0.076
Hb	123.12 ± 15.22	117.03 ± 19.75	1.439	0.155
ALB	37.06 ± 4.41	38.87 ± 6.11	-1.410	0.163
LDH	251.64 ± 62.67	251.26 ± 88.80	0.020	0.984
HDL-C	1.23 ± 0.30	1.07 ± 0.25	2.535	0.014
ATL	11.21 ± 8.66	14.43 ± 10.26	-1.291	0.202

NLR (Neutrophil-to-Lymphocyte Ratio), PLR (Platelet-to-Lymphocyte Ratio), MLR (Monocyte-to-Lymphocyte Ratio), CD3(Cluster of Differentiation 3, %), CD4 (Cluster of Differentiation 4, %), CD8 (Cluster of Differentiation 8, %), NK (Natural Killer Cell, %), B cell (B lymphocytes, %),WBC(White Blood Cell, ×10⁹/L), Neu(Neutrophil Count, ×10⁹/L),Lym(Lymphocyte, ×10⁹/L), Hb(Hemoglobin, g/L),ALB (Albumin, g/L), LDH (Lactate Dehydrogenase, U/L),HDL-C(High-Density Lipoprotein Cholesterol, mmol/L), ATL(Activated T lymphocytes, %)

The analysis compared SDS and SAS scores of 33 lung cancer patients in Group N before and after anti-anxiety and depression treatment. The SDS score demonstrated a significant decrease from 42.64 ± 6.32 before treatment to 37.06 ± 8.34 after treatment, yielding a t-value of 4.928 and a P-value of 0.00. The SAS score exhibited a similar significant reduction from 50.48 ± 12.94 before treatment to 43.61 ± 13.47 after treatment, with a t-value of 5.731 and a P-value of 0.00. These findings indicate that the anti-anxiety and depression treatment correlated with decreased SDS and SAS scores among the study participants (**Table 5**).

**Table 5:** Comparison of SDS and SAS scores of 33 lung cancer patients in group N before and after anti-anxiety and depression treatment.

Groups	Before Treatment	After treatment	t	P
SDS Score	42.64 ± 6.32	37.06 ± 8.34	4.928	0.00
SAS Score	50.48 ± 12.94	43.61 ± 13.47	5.731	0.00

**Table 6** presents a comparative analysis of immune function parameters among 179 patients with anxiety and depression tumors, categorized according to NLR levels. The analysis revealed that CD3 levels were notably lower in the low NLR group (57.71 ± 11.56) compared to the high NLR group (63.87 ± 14.95), demonstrating statistical significance (t = -2.550, P = 0.012). CD4 levels exhibited a similar pattern, with significantly lower values in the low NLR group (35.08 ± 11.10) compared to the high NLR group (42.68 ± 12.90), (t = -3.343, P = 0.001). Conversely, CD8 levels were significantly elevated in the low NLR group (26.84 ± 10.46) compared to the high NLR group (21.97 ± 9.57), (t = 2.631, P = 0.010). The CD4/CD8 ratio showed significantly lower values in the low NLR group (1.30 [1.07, 2.38]) compared to the high NLR group (2.00 [1.43, 3.10]), (Z = -3.138, P = 0.002). NK cell and B cell levels showed no significant differences between the groups. The study utilized a median NLR value of 3.24 as the grouping criterion.

**Table 6:** Effect of NLR on immune function in 179 patients with anxiety and depression tumors.

Groups	Low NLR group	High NLR group	t/Z	P
CD3	57.71 ± 11.56	63.87 ± 14.95	-2.550	0.012
CD4	35.08 ± 11.10	42.68 ± 12.90	-3.343	0.001
CD8	26.84 ± 10.46	21.97 ± 9.57	2.631	0.010
CD4/CD8	1.30(1.07,2.38)	2.00(1.43,3.10)	-3.138	0.002
NK	19.64 ± 10.02	19.10 ± 10.67	0.288	0.774
B cell	11.02 ± 5.71	9.82 ± 7.76	0.958	0.340

The median NLR value of 3.24 was used as the grouping standard.

## Discussion

Research indicates that cancer patients frequently experience various psychological challenges, particularly anxiety and depression, which affect both their quality of life and potentially their treatment outcomes (16, 17). The prevalence of anxiety and depression among cancer patients substantially exceeds that of the general population (18, 19). Research demonstrates that anxiety and depression in cancer patients can result in decreased appetite, nutritional deficiencies, and impaired sleep quality, creating a cycle that intensifies these psychological conditions and diminishes immune function, including CD8, CD4, and NK cells (6, 7, 20). These psychological disturbances may compromise treatment adherence, subsequently affecting therapeutic efficacy. Anxiety and depression markedly influence the immune function of cancer patients, with affected individuals showing notably reduced levels of CD3 and CD4 cells compared to patients without these conditions, while CD8 cell levels tend to be elevated (7). These immunological alterations may adversely influence disease progression and treatment effectiveness. Psychological interventions demonstrate considerable efficacy in alleviating anxiety and depression symptoms in cancer patients (21). Evidence suggests that targeted psychological interventions, including cognitive-behavioral therapy, pharmacological treatment, and social support networks, can substantially reduce anxiety and depression levels, enhance quality of life and treatment compliance, ultimately improving therapeutic outcomes and prognosis (22). Given the high occurrence of anxiety and depression in cancer patients and their negative impact on immune function and treatment outcomes, implementing active psychological interventions can effectively enhance patients’ mental health status and treatment results. Consequently, prioritizing patients’ mental health remains essential throughout cancer treatment (23).

In this retrospective study, no statistically significant differences in gender, age, tumor type, and stage were observed among the four groups: A (non-anxious and non-depressed), B (depression only), C (anxiety only), and D (anxiety and depression) (P ≥ 0.05), demonstrating their comparability. The body’s primary anti-tumor immune response relies on cellular immunity, where an elevated CD4+/CD8+ ratio indicates stronger anti-cancer immune capability, while a lower ratio suggests diminished immunity. Advanced hepatocellular carcinoma patients experiencing both anxiety and depression demonstrated significantly lower levels of T cell subsets and natural killer cells compared to non-anxious and non-depressed patients and those with isolated anxiety or depression, indicating that anxiety and depression may compromise cellular immune function (24). Research has shown that depression and anxiety levels influence the immune function of malignant cancer patients through various mechanisms (14). However, additional studies revealed that malignant tumor patients with depression exhibited increased inflammatory cytokines such as CRP, which enhance immunity, particularly in gastrointestinal cancer patients (5, 20). An elevated NLR correlates with increased levels of inflammatory cytokines (5). The present study revealed that patients with combined anxiety and depression, isolated anxiety, isolated depression, and neither condition showed significantly higher levels of CD3, CD4, CD8, NK cells, and CD4/CD8, and lower NLR compared to other groups, with statistical significance (P = 0.044, P = 0.001, P = 0.022, P = 0.039, P = 0.007, and P = 0.003). Tumor patients with anxiety and depression exhibited significantly lower CD3 and CD8 levels compared to non-anxious and non-depressed patients and those with depression alone (P = 0.005 and P = 0.037; P = 0.006 and P = 0.007). These findings suggest that negative emotional states such as anxiety and depression impair the immune function of tumor patients and may accelerate tumor progression.

Tumor patients with depression exhibit lower serum albumin levels compared to patients without anxiety and depression; similarly, tumor patients with both anxiety and depression demonstrate lower albumin levels compared to non-anxious and non-depressed patients, with statistical significance (P = 0.016 and 0.015). Studies indicate that psychological behavioral interventions improve patient appetite, which is crucial for the quality of life and prognosis of tumor patients experiencing anxiety and depression (5). This is particularly significant given that these patients often present with poor appetite and gastrointestinal dysfunction, contributing to reduced serum albumin levels.

Research has demonstrated that depression can reduce NK cell activity in patients with malignant tumors (25). The present study’s results indicate that patients with anxiety and depression exhibit significantly lower NK cell counts compared to non-anxious, non-depressed tumor patients, with statistical significance (P = 0.018). This suggests that anxiety and depression inhibit the nonspecific immune response of tumor patients to a measurable degree, potentially exacerbating their condition. While immune dysfunction represents a crucial pathogenic mechanism in tumor patients, the precise underlying mechanisms warrant further investigation. Both the current observations and related literature (26) indicate that anxiety and depression occur more frequently in cancer populations compared to the general population. Given that depression can compromise immune function, addressing patients’ psychological well-being may substantially improve their immune function. Unlike SSRIs/SNRIs, which primarily modulate serotonin, olanzapine’s dopamine D2 and 5-HT2A receptor antagonism may suppress stress-induced catecholamine release, reducing myeloid-derived suppressor cell (MDSC) recruitment and enhancing T/NK cell activity. This mechanism distinguishes it as a candidate for adjunctive immunotherapy (20, 27).

This study demonstrated that the control group (M group) and treatment group (N group) of lung cancer patients exhibited no statistically significant differences in gender, age, tumor type, and stage (P ≥ 0.05). The anxiety and depression assessments between the control and treatment groups (before drug treatment) showed no statistical significance (P = 0.385 and P = 0.603), establishing comparability between the groups. Since both groups comprised oncology patients naturally admitted to the hospital within six months, the control and treatment groups (before drug treatment) demonstrated substantial homogeneity. The treatment group exhibited significantly elevated levels of CD3, CD4, and NK cells compared to the control group (P = 0.001, P = 0.001, P = 0.001). Conversely, the control group demonstrated significantly higher NLR and B lymphocyte levels compared to the treatment group (P = 0.00 and P = 0.036). The treatment group also displayed higher levels of high-density lipoprotein, which was statistically significant (t = -2.535 and P = 0.014). These findings suggest that the antidepressant drug olanzapine may enhance cellular immunity in lung cancer patients with anxiety and depression (28). Olanzapine demonstrates efficacy in reducing cancer patient pain intensity, decreasing opioid use, improving cognition, and exhibiting anxiolytic effects. The treatment group showed a significant decrease in SDS after olanzapine treatment compared to pre-treatment levels (42.64 ± 6.32 VS 37.06 ± 8.34, t = 4.928, P = 0.00). Similarly, the treatment group demonstrated a significant reduction in SAS after olanzapine treatment compared to pre-treatment levels (50.48 ± 12.94 VS 43.61 ± 13.47, t = 5.731, P = 0.00). These results indicate that olanzapine, as an antidepressant, can effectively alleviate anxiety and depression levels in cancer patients (11, 29). The group with higher NLR compared to the lower NLR group showed significantly elevated values of CD3, CD4, and CD4/CD8 ratio (P = 0.012, P = 0.001, P = 0.002). Additionally, the higher NLR group demonstrated significantly lower CD8 values compared to the lower NLR group (Z = -3.138 and P = 0.002). While the NLR serves as an inflammatory marker, with studies suggesting that higher NLR correlates with poorer long-term prognosis in patients (30), the present research results indicate that higher NLR levels may stimulate cellular immunity in lung cancer patients. Several longitudinal studies indicate that, in the absence of targeted psychological or pharmacological interventions, anxiety and depression levels in many cancer patients tend to remain relatively stable situation (31), justifying Group M as a valid control.

### Clinical implications

The sustained nature of this stimulation and its potential adverse effects on patient immunology warrant further investigation (14). Additional research is necessary to elucidate the impact of depression on cancer patients and its underlying mechanisms, with the goal of developing more effective personalized treatments and improving overall patient outcomes (32, 33). Furthermore, the potential expanded application of olanzapine beyond its current antiemetic use merits investigation, particularly regarding its capacity to enhance both mental health and immune function in cancer patients. This research may enhance clinicians’ awareness and management of anxiety and depression in cancer patients, contributing to meaningful improvements in their quality of life.

### Study limitations

While this study provides valuable insights, several limitations warrant acknowledgment. The small sample size and lack of stratification by pathology/stage reduce statistical power and generalizability. Larger, stratified prospective trials are essential to validate these preliminary findings. This study did not assess overall or progression-free survival. However, elevated CD3/CD4 and NK-cell counts, together with a low NLR, may indicate improved clinical outcomes in lung cancer (34, 35). Future prospective RCTs must include survival endpoints to confirm clinical benefit of olanzapine-augmented immunotherapy. Chemotherapy regimens were not uniformly documented and could not be adjusted for, potentially introducing residual confounding despite balanced baseline characteristics (36–38).

## Conclusion

Cancer patients without anxiety or depression demonstrate stronger immune responses and superior nutritional status compared to those experiencing anxiety and/or depression. Research indicates that anxiety and depression specifically impair immune function in patients with malignant cancer. Studies show that olanzapine, an anti-anxiety and antidepressant medication, enhances immune function in lung cancer patients while simultaneously reducing their anxiety and depression symptoms.

## Data Availability

The raw data supporting the conclusions of this article will be made available by the authors, without undue reservation.
